# The Serological and Virological Investigation of Canine Adenovirus Infection on the Dogs

**DOI:** 10.1155/2013/587024

**Published:** 2013-09-24

**Authors:** Oya Bulut, Orhan Yapici, Oguzhan Avci, Atilla Simsek, Kamil Atli, Irmak Dik, Sibel Yavru, Sibel Hasircioglu, Mehmet Kale, Nuri Mamak

**Affiliations:** ^1^Department of Virology, Faculty of Veterinary Medicine, University of Selcuk, 42075 Konya, Turkey; ^2^Department of Virology, Faculty of Veterinary Medicine, University of Kyrgyzstan-Turkey Manas, Bishkek 720044, Kyrgyzstan; ^3^Department of Virology, Faculty of Veterinary Medicine, University of Mehmet Akif Ersoy, 15100 Burdur, Turkey; ^4^Department of Internal Medicine, Faculty of Veterinary Medicine, University of Mehmet Akif Ersoy, 15100 Burdur, Turkey

## Abstract

Two types of Canine Adenovirus (CAVs), Canine Adenovirus type 1 (CAV-1), the virus which causes infectious canine hepatitis, and Canine Adenovirus type 2 (CAV-2), which causes canine infectious laryngotracheitis, have been found in dogs. In this study, blood samples taken from 111 dogs, which were admitted to the Internal Medicine Clinic of Selcuk University, Faculty of Veterinary Medicine, with clinical symptoms. Seventy-seven dogs were sampled from Isparta and Burdur dog shelters by random sampling, regardless of the clinical findings. Dogs showed a systemic disease, characterized by fever, diarrhea, vomiting, oculonasal discharge, conjunctivitis, severe moist cough, signs of pulmonary disease and dehydration. Two dogs had corneal opacity and photophobia. In serological studies, 188 serum samples were investigated on the presence of CAV antibodies by ELISA. Total 103 (103/188–54.7%) blood samples were detected to be positive for CAV antibodies by ELISA. However, 85 (85/188–45.2%) blood samples were negative. Blood leukocyte samples from dogs were processed and inoculated onto confluent monolayers of MDCK cells using standard virological techniques. After third passage, cells were examined by direct immunoflourescence test for virus isolation. But positive result was not detected. In conclusion, this study clearly demonstrates the high prevalence of CAV infection in dogs.

## 1. Introduction

Canine adenovirus (CAV) can be grouped into two distinct but related serotypes, CAV-1 and CAV-2, based on serological tests [1] and molecular analyses [2–4]. Two types of Canine adenovirus (CAVs), Canine Adenovirus type 1 (CAV-1), the virus which causes infectious canine hepatitis, and Canine Adenovirus type 2 (CAV-2), which causes canine infectious laryngotracheitis, have been found in dogs [5]. CAVs belong to the genus *Mastadenovirus* of the family Adenoviridae. Virus enters the host via direct contact with contaminated saliva, urine, and faeces [6]. The incubation period is 4–7 days. CAV-1 replicates in vascular endothelial cells and causes a generalized infection characterized by hepatitis [7], whereas CAV-2 has an affinity for respiratory tract epithelium and is mainly associated with outbreaks of respiratory disease in kenneled dogs [8]. CAV-1 causes fever, often above 40°C, apathy, anorexia, abdominal pain, blood in faeces, acute/chronic hepatitis and interstitial nephritis, tenderness, vomiting, and diarrhoea. Dogs may develop bronchopneumonia, conjunctivitis, photophobia, and a transient corneal opacity, “blue eye”, which may occur after clinical recovery as result of anterior uveitis and oedema [9, 10]. CAV-2 is characterized by respiratory disorders, with clinical signs that include tonsillitis, pharyngitis, tracheitis, and bronchitis [11–13]. Confirmation of diagnosis and identification of CAV-1 and CAV-2 infections are usually based on virus isolation, electron-microscopic observation and serological tests [14]. There are distinct differences in structure, antigenicity, and pathogenicity between the two CAVs. Serological tests such as haemagglutination inhibition (HI), serum neutralization (SN), and enzyme-linked immunosorbent assay (ELISA) have been used detection of CAVs [15, 16]. The ELISA was found to be a highly efficient and rapid test to determine the immune status of dogs to infectious canine hepatitis virus and canine adenovirus type 2 [15]. The ELISA is a sensitive, reliable and fast method for the detection of anti-adenovirus antibody [16]. When compared with SN test, the ELISA has several advantages. It does not require cell culture, the risk of contamination is less and the optimization is easier than other serological test methods. With the advent of molecular techniques, restriction endonuclease analysis (REA) of the viral genome has been said to differentiate between the two viruses [11, 14, 17].

The objective of this study was to determine the presence of antigen and the prevalence of CAV type 1 and 2 exposure in shelter-housed and household dogs in several regions of Turkey. 

## 2. Material and Methods 

### 2.1. Study Population and Sample Collection

**Figure 1:**
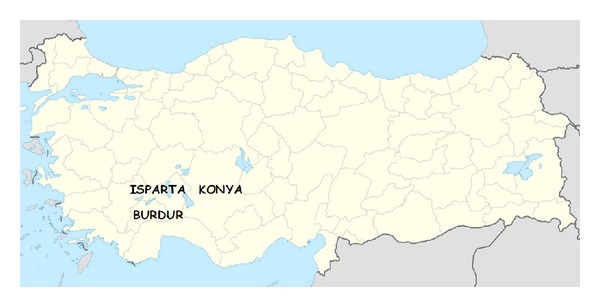
Sampling the provinces.

In this study, blood samples taken from 111 dogs, which were admitted to the Internal Medicine Clinic of Selcuk University, Faculty of Veterinary Medicine, with clinical symptoms, and from 77 dogs, which were housed at dog shelters in Isparta and Burdur provinces and were randomly sampled irrespective of their displaying clinical signs, were used (Figure 1, Table 1).

Of the animals that were sampled in the study, 108 were females and 80 were males. Seven of the animals were below 1 year of age, 53 were aged 1-2 years, 58 were 2 years of age, 64 were 3 years of age, 4 were 4 years of age, and 2 were 5.5–6 years of age (Table 2). Complete blood counts were performed for all dogs, and the presence of haematological disorders was recorded for each animal.

### 2.2. Cell Cultures

Madin-Darby Canine Kidney (MDCK) cells were grown in Dulbecco's Minumum Essential Medium (DMEM) supplemented with 100 U of penicilin/mL, 100 *μ*g of streptomycin/mL, and 10% calf serum for virus isolation attempts. The cells were incubated at 37°C in a 5% CO_2_ incubator. 

### 2.3. Blood Samples

Dog blood samples were drawn into normal tubes for serum and into EDTA tubes for the determination of CAV antibodies/antigens. Blood samples which were taken into normal tubes were centrifuged at 2000 rpm/15 min. The serums obtained were kept in deep freezer under −20°C. Serum samples were inactivated in 30 min at 56°C before being used. Leukocyte samples were prepared from blood samples taken into tubes with EDTA by a standard method. The leukocyte samples were kept in deep freezer under −20°C until being used.

### 2.4. Virus Isolation from Cell Culture

The leukocyte samples were retrieved from the deep freezer in which they were stored (−20°C) and rapidly thawed in a water bath at 37°C. The Madin Darby Canine Kidney (MDCK) cell culture was prepared in 96-well microplates, and 2 wells allocated for each serum sample were inoculated with 10 *μ*L of the leukocyte samples. Subsequently, the microplates were maintained at 37°C in an incubator with 5% CO_2_. The second passages of the samples grown in MDCK cell culture were made in 24 well-microplates and stored until being used for the immunofluorescence test. 

### 2.5. Immunofluorescence Test

For the conduct of the immunofluorescence test, the leukocyte samples inoculated into the 24-well microplates were retrieved from the deep freezer in which they were stored and thawed. Each well of the Lab-Tek chamber slides procured from Thermo Fisher Scientific (USA, catalogue no. 178599) was inoculated with 200 *μ*L of MDCK cells that were diluted with DMEM + 10% calf serum, such that a cell concentration of 1 × 10^5^ cells/mL was obtained. Next, the slides were incubated at 37°C for 24 hours. Later, 20 *μ*L of the second-passage fluid of each leukocyte sample was inoculated into two wells. After a 24-hour period allowed for adsorption, the cell surfaces were rinsed with phosphate buffer solution (PBS) and each well was added 200 *μ*L of virus growth medium. At the end of the third day, the medium was removed with the aid of a Pasteur pipette after the cell surfaces were rinsed with PBS, and then the cells were fixed in acetone for 10 minutes. In a dark environment, all wells were added 75 *μ*L of the ready-to-use CAV conjugate procured from VMRD (USA), and the slides were incubated at 37°C in a humid incubator for 30 minutes. Following incubation, the conjugate was removed from the wells, and the cells were rinsed minimum three times with FA Rinse Buffer, pH 9.0 (VMRD catalogue no. 210-90-RB) and allowed 10 minutes for drying. A droplet of 90% glycerol solution was added to each well. Finally, the wells were examined under a fluorescence microscope (Olympus Bx51, Japan). 

### 2.6. ELISA (Enzyme-Linked Immunosorbent Assay)

Canine adenovirus indirect ELISA Kit (EVL/European Veterinary Laboratory-Netherlands, catalogue no. D1003-AB01) was used for detecting CAV antibodies in dogs. The test was performed as per the manufacturer's instructions. The plates were then read on an automatic plate reader at 450 nm. 

### 2.7. Statistical Analysis

Seropositivity ratios were evaluated by *χ* ^2^ test (Minitab 12.0). *P* < 0.05 value was taken to indicate statistical significance.

## 3. Results 

Blood serum samples belonging to 188 dogs, which had either been admitted to the Internal Medicine Clinic of Selcuk University, Faculty of Veterinary Medicine, with clinical symptoms or had been sampled at the dog shelters they were cared after in Isparta and Burdur provinces, were examined using the ELISA method. Of these samples, 103 (54.7%) were found to be positive for antibodies against CAV infection (Table 3).

Of the 108 female animals sampled in the study, 55 (50.9%) were determined to be positive for CAV antibodies, while 48 (60%) of the sampled 80 male animals were confirmed to be positive (Table 3). Of the 7 animals below 1 year of age, only 1 (14.2%; 2-month-old female puppy) was positive, and the remaining ones were found to be negative for CAV antibodies. Of the 53 animals aged 1-2 years, 22 (41.5%); of the 58 animals aged 2 years, 31 (53.4%); of the 64 animals aged 3 years, 44 (68.7%); and of the 6 animals aged >4 years, 5 (83.33%) were found to be positive (Table 2).

Blood leukocyte samples from dogs were processed and inoculated onto confluent monolayers of MDCK cells using standard virological techniques. The inoculated cells were incubated at 37°C and observed daily for the appearance of cytopathic effect (CPE). After third passage, cells were examined by immunofluorescence test for virus isolation. No morphological changes were observed in cell cultures, and a positive result was not detected by immunofluorescence test.

*Clinical Findings*. Blood samples were taken from 111 dogs showing clinical symptoms which were brought to the Internal Medicine Clinic of Selcuk University, Faculty of Veterinary Medicine. Seventy-seven dogs were sampled from Isparta and Burdur dog shelters by random sampling, regardless of the clinical findings. Dogs showed a systemic disease, characterized by fever, diarrhea, vomiting, mucopurulent oculonasal discharge, mucopurulent conjunctivitis, severe moist cough, signs of pulmonary disease, and dehydration. Corneal opacity and photophobia were determined for two dogs.

## 4. Discussion

Despite the frequent occurrence of Canine Adenovirus worldwide, in Turkey, the only notified cases of Canine Adenovirus (CAV) infection are those reported by Okuyan [18] and Gür and Acar [16]. The antibody prevalence of the infection is reported to vary between 30 and 82% worldwide. Gür and Acar [16] reported that out of 94 dogs belonging to the Kangal, Turkish Greyhound, and Akbaş breeds, which were sampled in Konya and Eskişehir provinces, 82 (82.7%) were positive for CAV antibodies. 

Although, in most cases, CAV-1 and CAV-2 infections are not difficult to discriminate clinically from each other, they have the same morphological features under the electron microscope and the same cytopathogenic effects on cell cultures. There have been reports that CAV-2 can also infect the intestinal tract, one of the major target organs for CAV-1 [2, 19]. Diagnosis of CAV infections is usually based on serological tests, virus isolation, and negative staining [14].

In this study, the blood samples which are inoculated into cells were examined by direct immunofluorescence test for virus isolation. Viruses were not isolated from blood samples by direct immunofluorescence test. The adenovirus replicates in the lymph tissue and then spreads into the bloodstream. The replication reaches peak levels in 3–6 days after infection. Viral load decreases rapidly with respect to antibody production and longer CAV-2 cannot be isolated after 9 days [9, 20]. In the present study, the reason for unavailability of virus isolation may be due to the time of sampling. 

The CAV-2 is a highly contagious viral agent that is incriminated in canine respiratory tract disease, particularly in young dogs kept in a crowded environment, such as pet stores, boarding kennels, and veterinary hospitals. This classical syndrome is commonly referred to as kennel cough. Infection with CAV-2 is generally transient and seldom fatal, unless it is complicated with a secondary bacterial bronchopneumonia [21, 22]. 

As adenoviruses do not have a lipid envelope, they are very resistant to environmental conditions and maintain their viability for an extended period in the external environment. Animals that recover from adenoviral infection continue to shed the virus for an extended period of time, and it has been even reported that vaccinated animals also shed the virus. Therefore, the prevalence of CAV infection has been reported to be rather high in dog shelters that lack reliable vaccination records [23].

CAV-1, which causes infectious canine hepatitis (ICH), is eliminated from the body of infected animals by saliva, urine, and faeces and is transmitted to susceptible animals by direct contact with contaminated material. In young animals, CAV-1 causes serious clinical symptoms, including anorexia, ataxia, and paralysis, which may result in death; and compared with older animals, the clinical course of the infection is more severe in the young [6]. In the present study, antibody prevalence was found to be higher in animals aged 2 years and above. In this study, out of the 60 animals below 2 years of age, 23 (38%) and out of the 128 animals aged 2 years and above, 80 (62.5%) were confirmed to be positive for CAV antibodies. In particular, puppies below the age of 1, even if equipped with passive immunity through maternal antibodies, are not able to be protected against CAV infection when exposed, and mortalities may occur. Clinical ICH infection is more severe in young canids, as compared with adults [24]. 

The prevalence of the disease being higher in older animals that survive could be attributed to the high mortality in the young. In this study, few animals below 1 year of age were able to be sampled. Of the very few that were sampled, only 1 (a 2-month-old puppy) was determined to be positive for CAV antibodies. It was considered that this puppy had been protected by maternal immunity in early life. The remaining animals either could not have been exposed to the virus during life or could have suffered from low antibody levels in early life. No information was able to be obtained on whether the other animals survived. 

In a study conducted in jackals in California, Cypher et al. [25] determined that the prevalence of CAV antibodies was higher in adult animals. Higher CAV antibody prevalence among older age classes may be a function of greater mortality among pups resulting in a lower proportion of seropositive survivors in younger age classes [25, 26].

In Turkey, dogs both in rural areas and in dog shelters are not able to be regularly vaccinated against CAV-1 and CAV-2. In the present study, dogs that were admitted to the Internal Medicine Clinic of Selcuk University, Faculty of Veterinary Medicine, with complaints including fever (above 40°C), coughing, nasal changes, mucopurulent conjunctivitis, listlessness, inappetence, weight loss, pain and sensitivity of the abdominal region, vomiting, and diarrhea were sampled. Of the 111 dogs, which were admitted to the clinic, 37 were owned animals tended to by their owners. Twelve owned animals which were declared to have been vaccinated according to anamnesis were found to be negative for CAV antibodies. No reliable information was able to be accessed for the vaccination status of the remaining animals. Similarly, no reliable information was available for the vaccination status of the 77 dogs, which were sampled at the dog shelters in Isparta and Burdur provinces. The blood parameters of the animals that were admitted to the Internal Medicine Clinic of Selcuk University, Faculty of Veterinary Medicine, revealed the presence of lymphocytosis in some of the animals (5.84–12.61 m/mm^3^, reference range for dogs 0.6–5.1 m/mm^3^) and leucopenia (2.2–4.6 m/mm^3^, reference range for dogs 6.0–17.0 m/mm^3^) and anemia (0.99–5.24 m/mm^3^, reference range for dogs 5.5–8.5 m/mm^3^) in some others. Based on these findings, viral infection was suspected and the animals were tested for CAV. Two of the dogs exhibited photophobia and corneal opacity. In these 2 animals, although the presence of CAV viral antigen was not detected, the presence of CAV antibodies was confirmed. It is considered that these 2 unvaccinated animals were exposed to the disease and managed to survive the infection. For, in general, 7–10 days after being exposed to CAV, the acute signs of infection are replaced by corneal edema, which presents with a blue and rather opalescent appearance of the cornea. This appearance, which is generally observed in the convalescence period, disappears spontaneously. In some cases of mild infection, no clinical symptom is observed other than corneal edema. Although, rarely, this clinical picture may also develop as a vaccination complication, this option was discarded as the 2 animals that presented with corneal edema were unvaccinated. This clinical picture, specifically referred to as “Hepatitis Blue Eye” is known to be caused by CAV-1. However, as early detection was not possible, the CAV antibodies were not able to be typed in the animals that presented with leukopenia and lymphopenia. 

According to the vaccination schedule applied in Turkey, new-born puppies are vaccinated as from 2 months of age, 3 times at 21-day intervals with live multivalent vaccines (CAV-2, Distemper, Parvovirus, Parainfluenza, and Leptospira). Literature reports are available, which suggest that canine adenovirus antibodies produced against the two different CAV types provide cross-protection, owing to the antigenic similarity of CAV-1 and CAV-2 [19]. 

In the present study, apart from the correlation between age and antibody prevalence, the correlation between sex and antibody prevalence was also investigated. Accordingly, it was determined that antibody prevalence was higher in females (41%) in comparison with males (36%). Due to the number of female animals sampled in this study being greater than the number of sampled males, it is considered that the difference observed in antibody prevalence for sex is not significant (*P* > 0.05). On the other hand, in previously conducted studies, the correlation of sex with antibody prevalence was neither not investigated nor found to be statistically insignificant. Therefore, a comparative assessment was not able to be made in this study. 

Of the 188 animals sampled in the present study, the majority were unvaccinated dogs housed at shelters. In view of the animals that had been admitted to the internal medicine clinic of Selcuk University being vaccinated dogs, the antibody presence confirmed in these animals was considered as an indicator of the vaccination schedule having been properly applied in these animals. On the other hand, the antibody prevalence detected in the unvaccinated animals sampled at the dog shelters in Isparta and Burdur provinces was considered as an indicator of the presence of CAV infection in these dog shelters. This study clearly demonstrates the high prevalence of CAV infection in dogs, which live in groups and are not vaccinated on a regular basis. It is considered that regular vaccination would provide protection against the disease for a certain time period in dogs, and in particular in puppies, which live in groups under unfavorable conditions. Furthermore, as the transmission of the CAV occurs by environmental contamination (contact with infected faeces, urine, etc.), both the maintenance of hygiene conditions and the prevention of contact among dogs are of great importance in the control of the disease.

## Figures and Tables

**Table 1:** The animals according to province, breed, and sex.

Provinces	Breed	No. of samples	Sex Male/female		Total
Konya	Siberian Husky	2	—	2	111
	Crossbreed with Pitbull	2	1	1	
	Akbaş dog	1	—	1	
	Kars Sheeperdog	1	1	—	
	Crossbreed with Kangal	21	6	15	
	Spanish cocker	1	1	—	
	Pointer	1	—	1	
	Terrier	1	—	1	
	Beagl	1	—	1	
	Street dog	80	37	43	

Isparta	Street dog	49	19	30	49

Burdur	Street dog	28	15	13	28

Total		188	80	108	188

**Table 2:** Age distribution of dogs detected seropositive.

Ages	Number of dogs	Number of positives (%)
≤1 year	7	1 (14.2%)^c^
Between 1-2 years	53	22 (41.5%)^bc^
2 years	58	31 (53.4%)^ab^
3 years	64	44 (68.7%)^a^
>4 years	6	5 (83.33%)^a^

Total	188	103 (54.7%)

^a, b, c^values marked with different letters in the same line are statistically significant (*P* < 0.05, *X* ^2^ test).

**Table 3:** Results of CAV antibody status according to province, breed, and sex.

Provinces	Breed	No. of samples	No. of CAV antibody positive		Total (%)
			M	F	
Konya	Siberian Husky	2	—	1	68/111 (61.2%)^a^
	Crossbreed with Pitbull	2	1	1	
	Akbaş dog	1	—	1	
	Kars Sheeperdog	1	1	—	
	Crossbreed with Kangal	21	1	3	
	Spanish cocker	1	1	—	
	Pointer	1	—	1	
	Terrier	1	—	1	
	Beagl Limusin	1	—	—	
	Street dog	80	30	26	

Isparta	Street dog	49	9	14	23/49 (47%)^a^

Burdur	Street dog	28	5	7	12/28 (42.8%)^a^

Total		188	48 (60%)^A^	55 (50.9%)^A^	103 (%54.7)

^a, A^There is no statistically significant difference between provinces and sex.
